# Structure of Alloys for (Sm,Zr)(Co,Cu,Fe)_z_ Permanent Magnets: III. Matrix and Phases of the High-Coercivity State

**DOI:** 10.3390/ma14247762

**Published:** 2021-12-15

**Authors:** Andrey G. Dormidontov, Natalia B. Kolchugina, Nikolay A. Dormidontov, Mark V. Zheleznyi, Anna S. Bakulina, Pavel A. Prokofev, Aleksandr S. Andreenko, Yury V. Milov, Nikolay N. Sysoev

**Affiliations:** LLC “MEM”, 123458 Moscow, Russia; natalik014@yandex.ru (N.B.K.); ontip@mail.ru (N.A.D.); markiron@mail.ru (M.V.Z.); annbak@mail.ru (A.S.B.); pav3387@yandex.ru (P.A.P.); asa@phys.msu.ru (A.S.A.); milov.yv@mail.ru (Y.V.M.); nn.sysoev@physics.msu.ru (N.N.S.)

**Keywords:** Sm–Co permanent magnet alloys, magnetic hardening, high-coercivity structure, composition–structure–properties correlations

## Abstract

Observations of the surface domain structure (Kerr-effect), optical metallography, scanning electron microscopy (SEM-SE), and electron microprobe analysis (EPMA-SEM), measurements of major and minor magnetic hysteretic loops were used to study pseudo-single-crystal samples of (Sm,Zr)(Co,Cu,Fe)_z_ alloys subjected to heat treatments to the high-coercivity state, which are used in fabricating sintered permanent magnets. Correlations between the chemical composition, hysteretic properties, structural components, domain structure, and phase state were determined for the concentration ranges that ensure wide variations of 4f-/4d-/3d-element ratio in the studied samples. The phase state formed by collinear and coherent phase components determines the high coercive force and ultimate magnetic hysteresis loops of the pseudo-single crystals. It was found that the 1:5 phase with the hexagonal structure (P6/mmm) is the matrix of the alloys for (Sm,Zr)(Co,Cu,Fe)_z_ permanent magnets; the matrix undergoes phase transformations in the course of all heat treatments for the high-coercivity state. The heterogeneity observed with optical magnifications, namely, the observation of main structural components A and B, is due to the alternation, within the common matrix, of regions with modulated quasi-spherical precipitates and regions with hexagonal bipyramids (cellular phase) although, traditionally, many investigators consider the cellular phase as the matrix. It is shown that the relationship of volume fractions of structural components A and B that account for more than 0.9 volume fraction of the total, which is due to the integral chemical composition of the alloys, determines the main hysteretic performances of the samples. The Zr-rich phases, such as 5:19, 2:7, and 6:23, and a structural component with the variable stoichiometry (Sm(Co,Cu,Fe)_3.5–5_) that is almost free of Zr and contains up to 33 at% Cu, were found only within structural component A in quantities sufficient for EPMA analysis.

## 1. Introduction

Owing to the high both remanence and coercive force, samarium-cobalt- and neodymium-iron-boron-based permanent magnets draw the attention of numerous professionals.

In particular, considerable attention is paid to high-coercivity materials prepared from Sm–Co alloys containing copper, iron, and zirconium, the permanent-magnet properties of which are reached at the expense of the formation of nano-sized structure in the course of complex heat treatments, namely, high-temperature homogenizing annealing for supersaturated solid solution and subsequent long-term isothermal and stepped aging. As a result of purposeful alloying and heat treatments, the effective coercivity mechanism, namely, the domain-wall pinning at structural inhomogeneities and, therefore, the unique high time–temperature stability of magnetic properties of the (Sm,Zr)(Co,Cu,Fe)_z_-based permanent magnets were realized.

Despite numerous studies related to the formation of the structure and properties of (Sm,Zr)(Co,Cu,Fe)_z_ alloys and permanent magnets based on them, the phase composition, and sequence of the phase-formation and the description and sequence of individual phase reactions, which occur in the course of the complex heat treatment for the high-coercivity state, up to now are a matter of controversy of research groups of various scientific schools. It should be recognized that, currently, there is no consistently acknowledged concept on the formation of the high-coercivity structure of the (Sm,Zr)(Co,Cu,Fe)_z_ alloys. This fact restrains progress in developing and improving both new compositions of alloys for permanent magnets and technological procedures of their production.

Some of the most important data required for the objective evaluation of phase transformations in considered materials are the list and compositions of phases formed in (Sm,Zr)(Co,Cu,Fe)_z_ alloys after heat treatment for the high-coercivity state. At different times of investigations, this problem was studied and presented in corresponding reports [1,2,3,4,5,6,7,8].

According to these studies, the starting structural state of experimental samples was characterized by fine-grained structure, namely, these samples were either anisotropic samples of permanent magnets sintered from fine powders [2,3,4,5,6,8] or isotropic cast samples prepared by vacuum induction or arc melting followed by solidification in water-cooled copper crystallizer or mold [1,7]. In these studies, only Derkaoui et al. [1] used the heat treatment of samples for 800 h; the other authors, in order to study the phase composition of samples, used traditional heat-treatment conditions for the formation of high-coercivity state which are accepted in magnet production [2,3,4,5,6,7,8]. Thus, in the majority of studies, experimental samples initially were characterized by highly refined phase structures.

As a result of efficient homogenization, the subsequent phase transformations that occur during isothermal and stepped aging are difficult to specify in studying the material structure, even using up-to-date methods and equipment, because of nano-scaled structural changes. At the same time, results of phase reactions occurring upon heat treatment are clearly detected in changes of the structure-sensitive parameters of material, in particular, coercive force [1,2,3,4,5,6,7,8].

In our previous studies, we showed that samples prepared from grains separated from coarse-grained ingots of (Sm,Zr)(Co,Cu,Fe)_z_ alloys with the optimum chemical composition (pseudo-single-crystal samples), which were subjected to traditional heat treatment for the high-coercivity state, demonstrate ultimate hysteresis loops that are free of steps in the magnetization-reversal curves, which usually are observed for samples having heterogeneous morphology [9,10,11]. In our case, the structure of the material characterized by ultimate hysteresis loops is heterogeneous even at the optical-magnification scale and contains at least three structural components (A, B, and C); the quantitative relationships of the volume fractions of these structural components in the material strictly correlate with the hysteretic parameters of these samples. Thus, the detection of microstructure elements typical of the formed high-coercivity state of the alloys becomes easier.

The aim of the present study, which widens our earlier developments [9,10,11], was the comprehensive investigation of interrelations between the hysteretic properties and phase composition of pseudo-single-crystal (Sm,Zr)(Co,Cu,Fe)_z_ samples, in particular, to obtain knowledge about (1) the interrelation between the domain structure and morphology of fine structure of main structural components; (2) chemical composition of them and minor phases, listing and positioning them in the (Co,Cu,Fe)–Sm–Zr compositional triangle; and (3) structural component being the matrix in the course of all heat treatments for the high-coercivity state of the (Sm,Zr)(Co,Cu,Fe)_z_ alloys.

## 2. Materials and Methods

Experimental samples were prepared in accordance with the Sm_1−x_Zr_x_(Co_1−a−b_Cu_a_Fe_b_)_z_ stoichiometry using a vacuum induction melting of individual components in a high-purity argon atmosphere; the chemical compositions of the samples are given in Table 1. As the starting materials, Sm, Zr, Co, Fe, and Cu were used; the contents of base component in them are >99.9, >99.97, >99.98, >99.7, and >99.97 wt%, respectively.

The metals were melted and the melt was slowly cooled in Al_2_O_3_ crucibles that were thermally isolated from the water-cooled inductor. As a result, a coarse-grained ingot with a grain size of 4–6 mm was prepared. The chemical analysis of ingots was carried out by inductively coupled plasma optical emission spectrometry (ICP-OES) using dissolved samples.

Alloy ingots were separated into individual grains that were subjected to conventional heat treatment for the high-coercivity state in a high-purity argon atmosphere; during heat treatment, ingots were under the powdered alloy having the same composition, which was used as a getter. The heat treatment included the high-temperature solid-solution treatment at 1160–1180 °С for 5 h and subsequent quenching to room temperature, isothermal annealing at 800 °С for 20 h, and stepped aging from 800 °С to 400 °С with an average cooling rate of 100 °C/h.

The as-cast samples subjected to heat treatment to different stages were not single-phase and cannot be specified as single crystals. At the same time, the structure of Sm_1−x_Zr_x_(Co_1−a−b_Cu_a_Fe_b_)_z_ samples in the form of individual grains comprised collinear phases. In other words, the easy magnetization axes (EMA) of each of the phases within an individual-grain sample were parallel/antiparallel to each other, and, from the viewpoint of magnetic properties, the samples are characterized by properties typical of single crystals. Such samples are described as pseudo-single crystals.

The pseudo-single crystals subjected to different heat treatments were ground to form balls 2.5–3.5 mm in diameter using a special abrasive tool, and subsequently were used for measurements of major and minor magnetic hysteresis loops at room temperature in external magnetic fields H_MAX_ = ±30 kOe. The measurements were performed on a vibrating-sample magnetometer (VSM). Some measurements of magnetic properties of high-coercivity samples were carried out under analogous temperature conditions in magnetic fields H_MAX_ = ±90 kOe using VSM option of a PPMS-9T (QuantumDesign, San Diego, CA, USA) installation. 

Before magnetic measurements, the samples were magnetized with a pulsed magnetic field of 100 kOe 8 ms in pulse duration. The magnetic field amplitude was determined with a Mech-2 pulsed tesla-meter.

Studies of the domain structure and microstructure were performed for samples, the magnetic properties of which preliminarily were comprehensively characterized at room temperature. After recording major and minor hysteresis loops, samples were demagnetized at “0” by an oscillating external magnetic field variable-polarity with a decreasing amplitude.

Sections were prepared at prismatic and basal planes using standard procedures and diamond pastes. The canonical behavior of the major and minor hysteresis loops of the (Sm,Zr)(Co,Cu,Fe)_z_, pseudo-single-crystal samples is typical of materials with the domain-wall-pinning coercivity mechanism. This allowed us to study the domain structure transformation by Kerr-effect microscopy using a traditional optical microscope; samples were studied after action of external magnetic field of a given value.

The microstructure of basal and prismatic planes of the samples was studied after etching in reagents HNО_3_/C_2_H_5_OH = 1/99 and FeCl_3_/HCl/C_2_H_5_OH = 5/10/85 mas.%. The volume fractions of structural components were determined using a Thixomet Pro automated image-analysis software. The composition of structural elements was studied using a scanning electron microscope equipped with a wavelength-dispersive spectrometer (EPMA-SEM Camebax operated with SmLα, ZrLα, CoKα, FeKα, and CuKα characteristic lines) and ZAF correction; the fine structure was studied in secondary-electron mode. 

The electron-microscopic study of each of the samples was started with scanning the maximally possible section area to determine the integral chemical composition and check the correspondence between the sample composition and ICP-OES data. Individual structural elements were studied by (i) analogous scanning of the areas that were obviously far from interfaces, (ii) scanning of characteristics structural elements at points, and (iii) scanning across the boundaries of structural components. Pure metals were used as standards. The relative error of determination of concentrations (at%) did not exceed 0.5%, 1.0%, 4.0%, 2.0%, and 2.0% for Co, Fe, Zr, Sm, and Cu, respectively.

## 3. Results

**Figure 1:**
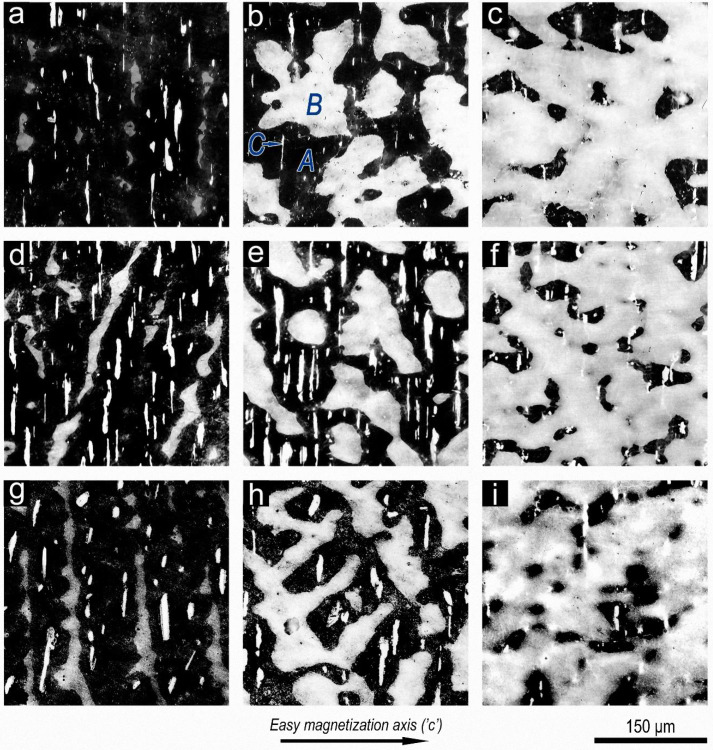
Typical microstructures of pseudo-single-crystal samples (etched) in the high-coercivity state: (**a**–**c**)—Sm_0.85_Zr_0.15_(Co_0.702_Cu_0.088_Fe_0.210_)_z_ (with z = (**a**) 6.0, (**b**) 6.5, and (**c**) 6.8); (**d**–**f**)—Sm_0.81_Zr_0.19_(Co_0.702_Cu_0.088_Fe_0.210_) _z_ (with z = (**d**) 6.0, (**e**) 6.3, and (**f**) 6.7), and (**g**–**i**)—Sm_0.85_Zr_0.15_(Co_0.665_Cu_0.075_Fe_0.260_) _z_ (with z = (**g**) 6.0, (**h**) 6.4, and (**i**) 6.7).

Figure 1 shows typical microstructures that demonstrate the first, coarsest, level of heterogeneity of the studied (Sm,Zr)(Co,Cu,Fe)_z_ samples in the high-coercivity state, which are characterized by different 4f-/4d-/3d-element relationships, namely, different proportions Sm/Zr/(Co,Cu,Fe) in the integral composition of the alloys. The microstructures of samples characterized by the same 4f-/4d-element relationship but differing in the Co/Cu/Fe relationship of 3d-elements are also shown.

The microstructures of high-coercivity samples of the Sm_1−x_Zr_x_(Co_0.702_Cu_0.088_Fe_0.210_)_z_ alloys, which are characterized by monotonous variations of Z ((4f-,4d-)/3d-element relationship), i.e., the alloys with the same relationship of 3d elements but with different 4f/-4d-element relationships, namely, with X = 0.15 and 0.19, are shown in Figure 1a–c and Figure 1d–f, respectively.

In turn, the microstructures of high-coercivity Sm_0.85_Zr_0.15_(Co_1−a−b_Cu_a_Fe_b_)_z_ samples, which are characterized by monotonous variations of Z ((4f-,4d-)/3d-element relationship) and the same relationship of 4f/4d elements (0.85/0.15) but by different relationships of 3d elements, namely, (Co_0.702_Cu_0.088_Fe_0.210_) and (Co_0.665_Cu_0.075_Fe_0.260_), are shown in Figure 1a–c and Figure 1g–i, respectively.

As is seen from Figure 1, the microstructure of all samples consists of three structural phase components (marked in Figure 1b); these are the dark-grey (A), dendritic-like, bright-grey (B), and anisotropic, almost-white (C) components; precipitates of component C are extended along the basal plane of sample and, for all samples, localized within component A. The relationships of volume fractions of all the structural components in samples Sm_1−x_Zr_x_(Co_1−a−b_Cu_a_Fe_b_)_z_ monotonically vary as the each of independent variables (X and Z) monotonically changes and dependent variables (a and b) mutually change. This is typical of all studied series of alloys (see Table 1).

The indicated changes in the volume ratios of structural components (A, B, and C), which are caused by changes in the 4f-/4d-/3d-element relationships in each of the experimental (Sm,Zr)(Co,Cu,Fe)_z_ series are accompanied by monotonic changes in their hysteretic characteristics.

**Figure 2:**
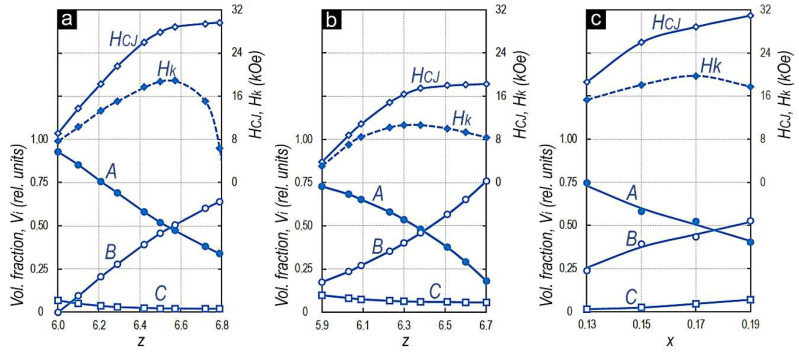
Diagrams plotted on coordinates Z (X) (chemical composition)—volume fractions of structural components A, B and C (Vi)—coercive force (H_CJ_), and hysteresis loop squareness parameter (H_k_) for the high-coercivity (**a**) Sm_0.85_Zr_0.15_(Co_0.702_Cu_0.088_Fe_0.210_)_z_, (**b**) Sm_0.85_Zr_0.15_(Co_0.665_Cu_0.075_Fe_0.260_)_z_, and (**c**) Sm_1-x_Zr_x_(Co_0.702_Cu_0.088_Fe_0.210_)_6.4_ samples.

These changes are given in Figure 2 in the form of three diagrams plotted on coordinates Z(X) (chemical composition)—volume fractions of structural components A, B, and C (V_i_)—coercive force (H_CJ_), and hysteresis loop squareness parameter (H_k_) (that corresponds to the external demagnetizing field in the second quadrant of the hysteresis loop, at which the remanence (4πJ_R_) of a sample decreases by 10% of the nominal value).

Figure 2a shows the corresponding diagrams for the high-coercivity samples prepared from the Sm_0.85_Zr_0.15_(Co_0.702_Cu_0.088_Fe_0.210_)_z_ alloy series, in which the content of 3d elements monotonically increases and the 4f-/4d-element relationship (Sm/Zr) is unchanged and equal to 0.85/0.15.

Figure 2b shows the analogous dependences for the Sm_0.85_Zr_0.15_(Co_0.665_Cu_0.075_Fe_0.260_)_z_ samples characterized by the same Sm/Zr relationship but the other relationship of 3d elements. Figure 2c shows the diagram for the different series of alloys, which are characterized by the same relationship of (4f/4d)/3d elements Z = 6.4 and monotonic variations of the 4f-/4d-element ratio, namely, for the Sm_1−__x_Zr_x_(Co_0.702_Cu_0.088_Fe_0.210_)_6.4_ alloys.

As the relative content (X) of 4d element (Zr) in the integral composition of the Sm_1−x_Zr_x_(Co_1−a−b_Cu_a_Fe_b_)_z_ alloy increases, the volume fraction of structural component C in all alloys increases. In this case, within each of the series, as the 3d-element content (Z) increases, the volume fraction of C component decreases.

Within each of the experimental alloy series, the volume fractions of structural components A and B, the sum of which corresponds to more than 0.9 volume fractions, monotonously change; in this case, the dominant volume fraction of structural component A changes to the dominant volume fraction of structural component B.

As the relative content of 3d elements (Z) in Figure 2a,b or 4f element (X) in Figure 2c increases and, therefore, the volume fraction of structural component B increases, an intense increase in the coercive force (H_CJ_) is observed until the alloys reach the compositions at which the equality of volume fractions of structural components A and B (V_A_ = V_B_) takes place.

After that (at V_A_ < V_B_), the increase in H_CJ_ becomes slower and the dependence goes to the “plateau”. In this case, the event corresponding to the equality of volume fractions of structural components A and B (V_A_ = V_B_) correlates with the extreme (maximum) in the dependence of the hysteresis loop squareness parameter on the alloy composition (H_k_ = f(Z,X)), i.e., the reaching of the chemical composition of alloys to the condition V_B_ > V_A_ is accompanied by worsening the hysteresis loop squareness; this is true for all (Sm,Zr)(Co,Cu,Fe)_z_ alloy series (for detail, see [9]).

**Figure 3:**
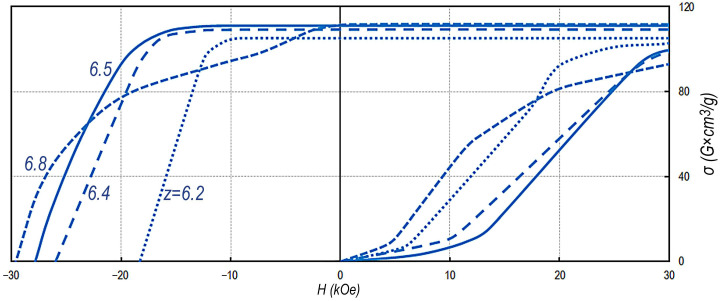
Magnetization curves from the state obtained upon demagnetization with an oscillating external magnetic field variable-polarity with a decreasing amplitude and demagnetization portions of major hysteresis loops of high-coercivity pseudo-single-crystal Sm_0.85_Zr_0.15_(Co_0.702_Cu_0.088_Fe_0.210_)_Z_ samples; numbers at the dependences correspond to *Z* values.

Samples with the chemical compositions corresponding to the condition V_A_ ≥ V_B_ demonstrate almost ultimate hysteresis loops. As an example, Figure 3 shows the demagnetization curves for the Sm_0.85_Zr_0.15_(Co_0.702_Cu_0.088_Fe_0.210_)_z_ alloy series.

The demagnetization curves given in Figure 3 clearly demonstrate that, in the case of dominant volume fraction of structural component A in samples (V_A_ ≥ V_B_), the demagnetization portions of the major hysteresis loops of the samples are characterized by equalities 4πJ_S_ = B_R_ and (BH)_MAX_ = (4πJ_S_)^2^/4, i.e., the obtained hysteresis loops are ultimate. However, as Z increases and results in the V_A_ < V_B_ relationship, the squareness of the hysteresis loop decreases and a “shoulder” appears in the demagnetization curves of the hysteresis loop; the value of the “shoulder” increases as the difference (V_B_ − V_A_) increases (for other alloy series, see [9,10,11]).

Note that, in Figure 3, the Sm_0.85_Zr_0.15_(Co_0.702_Cu_0.088_Fe_0.210_)_z_ samples that indicate almost ultimate regular hysteresis loops are obviously heterogeneous with optical magnifications (Figure 1a–c). In Figure 3, the heterogeneity manifests itself in the magnetization curves of samples from the state demagnetized with an oscillating external magnetic field variable-polarity with a decreasing amplitude.

**Figure 4:**
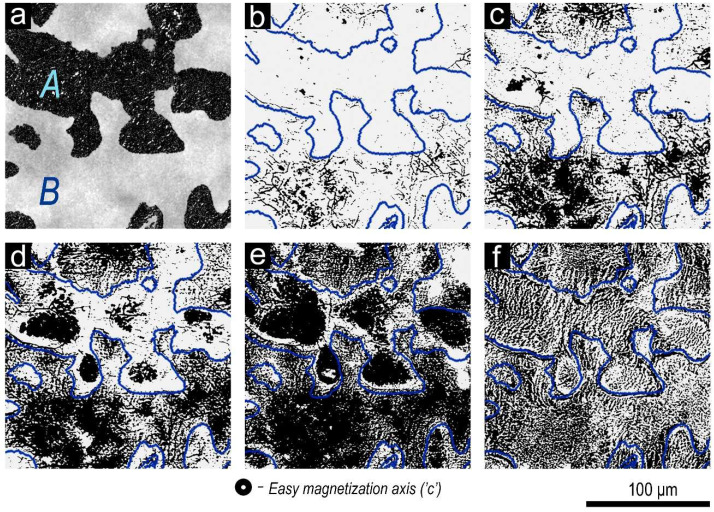
(**a**) Microstructure at the basal plane of the Sm_0.85_Zr_0.15_(Co_0.702_Cu_0.088_Fe_0.210_)_6.6_ sample and (**b**–**d**) morphology of domain structure (Kerr effect) for the same area upon progressive magnetization reversal from the magnetic saturation state with reverse magnetic fields of (**b**) 10, (**c**) 15, (**d**) 20, and (**e**) 30 kOe, and (**f**) domain structure of sample in the state after demagnetization with an oscillating external magnetic field variable-polarity with a decreasing amplitude.

After such a demagnetization, domain walls in all structural elements are in potential wells. In this case, as the external magnetized field increases, the magnetization curves of samples exhibit clear “bends” that are due to the relationships of volume fractions of structural components (A and B) and ranges of local coercivity of domain walls in the structural elements. Ranges of local coercivity are given in our study [10] along with the analysis of the morphology of the domain structure in structural components A and B upon magnetization reversal. The typical morphology and development of magnetic domains in structural components A and B of the pseudo-single crystals of high-coercivity alloy samples are cardinally different (Figure 4).

For illustration, Figure 4 shows images of the same field of view at the basal plane of the Sm_0.85_Zr_0.15_(Co_0.702_Cu_0.088_Fe_0.210_)_6.6_ sample; wide areas corresponding to structural components A and B are observed. In the case of this sample, the volume fraction of structural component B insignificantly exceeds that of structural component A (see Figure 4a).

The domain structure in structural components A and B develops upon magnetization reversal from the saturation state over wide magnetic field range. The primary reverse domains nucleate in central areas of both structural components and develop toward boundary areas.

Domains of structural component B and the transformation process of the domain structure in applying magnetic field are identical to those for (Sm,Zr)(Co,Cu,Fe)_z_ permanent magnets sintered from fine powders. The domain structure is characterized by the formation of reverse domains from an infinitely great number of nuclei upon magnetization reversal. Magnetic domains on the plane are fiber-like, zig-zagging in form, and are submicron in width that remains unchanged; each domain develops only along the length. As the negative field increases over a wide range of external fields, areas of structural component B are filled with reverse domains at the expense of newly formed domains and packing the reverse domain network.

The domain structure of component A differs qualitatively. It is more likely to be identical to that of quasi-binary Sm(Co,Cu)_z_ and Sm(Co,Cu,Fe)_z_ (z = 5–6) alloys [12]. The domain structure of component A upon magnetization reversal transforms from a limited number of nuclei and is characterized by the development of labyrinth domains that transfer to fernlike magnetic domains widening isotropically in all directions. This occurs in the narrower field range as compared to the field range for structural component B. The domain nucleation occurs also in central areas of component A and in areas adjacent to component C characterized by the lowest resistance to magnetization reversal.

The maximum local coercivity of domain boundaries was found for coring areas that transition from one structural component to another (A–B). This is clearly observed in comparing the topology demonstrated in panels in Figure 4, namely, boundary areas corresponding to structural components A and B in Figure 4a and wide areas that, after the action of a reverse field of 30 kOe, do not undergo magnetization reversal (Figure 4e).

The morphology of domain structure and character of its transformation in main structural component A and B indicate the different nature of domain-wall pinning partitions in them. This is confirmed by result of the study of fine phase structure.

**Figure 5:**
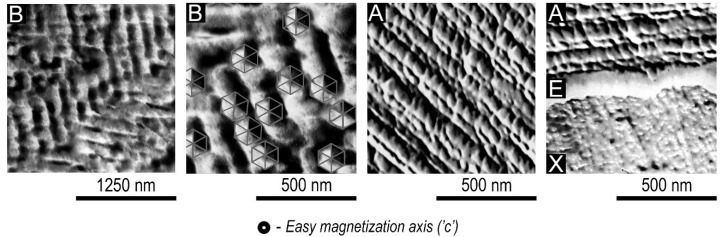
Typical morphology of structural components B and A and the E and X phases within component A (SE, SEM).

Figure 5 shows the typical morphology of structural components A and B (indicated in corresponding areas of image) at basal planes of high-coercivity samples.

The structure of component A is presented by regular rows of quasi-spherical precipitates less than 50 nm in size.

In turn, component B is characterized by a slightly coarser morphology that, on all grounds, is analogous to the known cellular structure of sintered (Sm,Zr)(Co,Cu,Fe)_z_ magnets. Precipitates in structural component B are arranged in chevrons made typically at 120° angles with a period of 120–140 nm. In this case, because of tilting a sample with respect to a gun and secondary-electron detector, the play of light and shade of the precipitates clearly indicates their characteristic faceting that often is diffuse as a result of etching (marked B in Figure 5).

About 30 high-coercivity samples differing in the composition, which correspond to the experimental series given in Table 1, were studied by EPMA-SEM.

It was found that, as the integral relationship of (3d)-to-(4f-4d) elements changes (Z), the samarium and zirconium concentrations in the main structural components remain almost unchanged, i.e., the (3d-)/(4f-4d-) element relationships in each of them (z_A_ and z_B_) are unchanged with changing the chemical composition within the experimental series.

In turn, the relationships of 3d elements in main structural components A and B in the samples vary within no more than 1.5–2.0 at% for experimental alloy series 1–4 (see Table 1) and within 3.0 аt% for series 5 and 6. It should be noted that changes in the copper and iron contents in components A and B mainly depend on the relationship of iron-to-zirconium contents. In particular, as Z of alloy of 5 and 6 series increases, the increase in the copper content and decrease in the iron content occur more dynamically and over a wider concentration range. (The chemical compositions of structural components of A and B in the high-coercivity Sm_1−X_Zr_X_(Co,Cu,Fe)_z_ alloys are described in detail in [10]).

In the majority of the studied samples, structural elements having signs of individual phases were found along with structural components A and B. Note that these additional phase components (marked as C, D, and E) are present only in structural component A. In this case, the formation of phases C, D, and E have their own peculiarities, i.e., in alloys with the dominant volume fraction of component A (V_A_ > V_B_), components C and E are often observed, whereas, in alloys with V_A_ < V_B_, mainly component D is observed.

Moreover, only within structural component A, structural element X having a variable composition is found; this, by all indications, results from the coalescence of precipitates forming the heterogeneity of this structural component. One of the peculiarities of structural element X, sizes of which allow us to use EPMA, is a thin rim of E phase. It is necessarily accompanying the X phase in the structure of experimental samples (Figure 5, right image).

**Figure 6:**
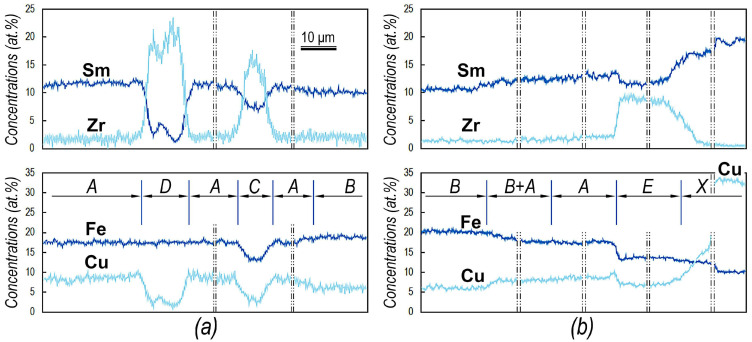
Element concentration distributions for structural components of high-coercivity pseudo-single-crystal (**a**) Sm_0.85_Zr_0.15_(Co_0.702_Cu_0.088_Fe_0.210_)_6.6_ and (**b**) Sm_0.87_Zr_0.13_(Co_0.690_Cu_0.070_Fe_0.240_)_6.5_ samples (SEM, EPMA).

Figure 6 shows the Sm, Zr, Fe, and Cu distribution curves for the structural elements in two (Sm,Zr)(Co,Cu,Fe)_z_ samples of alloys in high-coercivity states, which demonstrate the presence of all structural components and phases found in terms of the present study (except oxides, carbides, etc.).

In turn, for comparison, the two variants of averaged chemical compositions of structural components A and B, phases C, D, E, and component X, which are present only within component A, are given in Table 2.

Thus, the qualitative (morphology of magnetic domains) and quantitative (local coercivity of domain walls) differences in the magnetic domain structures of main structural components A and B can be explained by substantial differences in their nano-heterogeneous structure.

## 4. Discussion

### 4.1. On the Matrix

Fidler et al. [13], when studying Sm_2_Co_17_-based permanent magnets, stated that two types of precipitation hardened magnets can be distinguished; one of them contains 2:17 phase precipitates in a 1:5 matrix, and the other type of magnet forms 1:5 precipitates in a 2:17 matrix. In turn, Lefevre et al. [14], when optimizing the magnets based on investigations of quinary Sm–Co–Cu–Fe–Zr phase diagram, concluded that, upon the formation of high-coercivity structure, the “1/7 disordered structure is destroyed and the 2/17R phase nucleates and grows in the 1/5 matrix”.

When considering the genesis of cellular microstructure of Sm_2_(Co,Fe,Cu,Zr)_17_ permanent magnets, C. Maury et al. [15] stated that the considered phase transformation process appears as “ordering and precipitation from a supersaturated 1:7 matrix”; in turn, the “cells originate from the nucleation of R2:17 ordered domains”. In the course of formation of these cells, the excess of copper and samarium appears. This induces a related increase in the Cu and Sm contents in the surrounding matrix that tends towards 1:5 stoichiometry and forms the cell boundaries. Finally, according to Maury et al. [15], a minor part of remaining matrix enriched in Cu constitutes the cell boundaries.

However, it is more likely, owing to the stringency of assumptions of A.E. Ray [8], the majority of authors, for example, [1,3,5,6,7,16,17,18], when considering the structure of these materials in the high-coercivity state, indicate the 2:17R-based phase as the matrix phase. In our opinion, in such an approach, a case of transference “main phase” and “matrix” takes place, which results in a methodological error that makes difficult the understanding of the formation processes of the high-coercivity structural state of permanent magnets based on (Sm,Zr)(Co,Cu,Fe)_z_ alloys.

Indeed, the 2:17R-based phase in such sintered permanent magnets, which corresponds to the significant volume fraction of samples, mainly controls their high magnetization and Curie temperature and, with some reservations, can be determined as the main phase. However, this is insufficient in order to determine this phase as the matrix.

It is known that the supersaturated solid solution based on the TbCu_7_ disordered phase with a hexagonal structure (P6/mmm) is the main phase component of the alloy after high-temperature homogenization and quenching. Upon subsequent isothermal and stepped aging, structural transformations with the formation of final cellular structure occur in the solid solution. 

As a result of heat treatments, the structure of the alloy in the high-coercivity state consists of cells in the form of hexagonal bipyramids that are discretely distributed with a period of 60–200 nm in accordance with the alloy composition and heat treatment; the bipyramids are formed by the rhombohedral Th_2_Zn_17_ (R$\bar{3}$m) phase, and their height is along the easy magnetization axis.

The cells are separated by a continuous network of the phase that is coherent with the cell structure but has the hexagonal CaCu_5_-based structure (P6/mmm) and ensures the boundary between cells, which is 6–20 nm thick (boundary phase). The whole anisotropic cell-boundary massive, along the basal plane, is penetrated with thin extended Z-phase plates (lamellas) 2–4 nm thick, which are coherent, in turn, with cell and boundary phases.

The given description of the structure that results from the heat treatment is unlikely to cause questions. In this case, the conception “matrix phase of 2:17R cell” is equivalent to the statement that the matrix is discrete!

Already, J. D. Livingston and D. L. Martin [19] have focused on the contradiction. When they attempted to resolve it, they could only complicate it. They resumed: “This structure evolved by precipitation of ordered 17:2 particles from a disordered 5:1 matrix, and coarsens with aging until full coherence is lost and the cellular structure breaks up. The over-aged alloy consists of semi-coherent 5:1 particles in a 17:2 matrix, with a single set of interface dislocations accommodating the lattice mismatch.”

After such florid sentences, the thought of an ageless paradox involuntarily comes: what came first: the chicken or the egg?

The observations of the microstructures with electron microscopic resolutions, which were formed in pseudo-single crystals as a result of complex heat treatment, allow us to make the assumption with respect to the structural constitution of these samples.

We highlight that:

(i) samples, as follow from the analysis of domain, optical and electron micrographs, and magnetization curves as well, are characterized by significant heterogeneity as compared to that of traditional magnets sintered from fine powders;
(ii) in this case, the whole phase massive of pseudo-single crystals is ideally anisotropic; this is indicated by ultimate hysteresis loops of samples.

**Figure 7:**
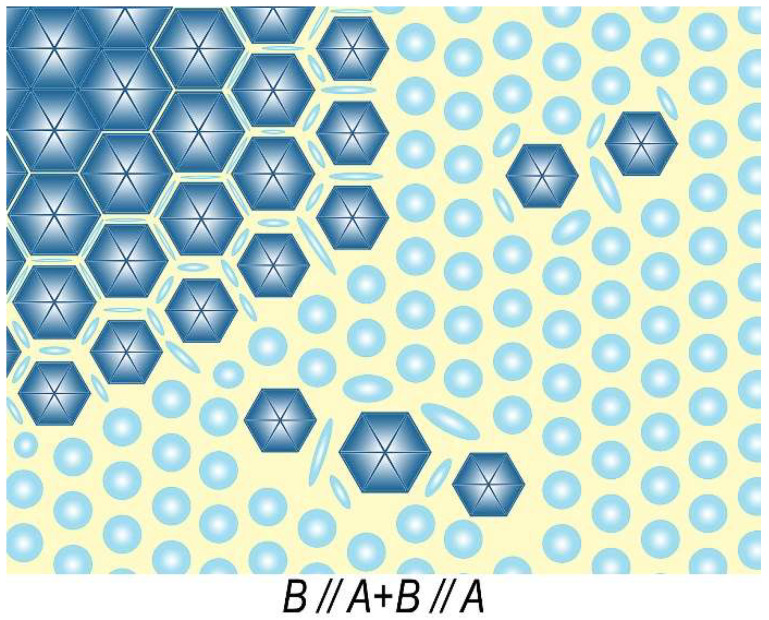
Appearance (schematic model) of typical microstructure on the basal plane of (Sm,Zr)(Co,Cu,Fe)z pseudo-single crystals in the high-coercivity state.

Figure 7 shows a model of typical microstructure observed, with electron-microscopic magnifications, on the basal plane of (Sm,Zr)(Co,Cu,Fe)_z_ pseudo-single crystals after heat treatment for the high-coercivity state.

The left upper angle of the scheme indicates structural component B; the bottom right angle corresponds to structural component A. The transition area (coring) A + B is between components A and B.

It seems that structural component A, in passing to component B, “flows” in spaces between hexagonal bipyramidal cells and forms the boundary structure. Upon the A–B transition, precipitates typical of component A remain but, near cells formed component B, deform and loss their quasi-spherical shape. From the periphery of B component area to its center, spaces between pyramidal cells decrease to minimum; this results in the aforementioned peculiarities of the formation of domain structures upon magnetization reversal of samples. The magnetization reversal of all pseudo-single crystals starts from the formation of reverse domains in the central areas of structural components A and B, whereas the maximum local coercivity of domain wall is typical of transition areas A + B (see Figure 4).

Thus, taking into account the above arguments, the hexagonal 1:5-based structure (P6/mmm) is the (Sm,Zr)(Co,Cu,Fe)_z_ permanent magnet matrix undergoing phase transformations throughout the heat treatment period for the high-coercivity state:

1. The disordered TbCu_7_-based solid solution (P6/mmm) formed in the course of homogenizing at 1160–1180 °С, owing to the high supersaturation and activation of diffusion, transforms at isothermal aging temperatures of 800–850 °C; the transformation is accompanied by ordering, deposition, and growth of discrete Th_2_Zn_17_ phase (R$\bar{3}$m) cells. Structural component B is formed by ensembles of these cells in the boundary matrix. The same matrix flows into structural component A, free of cells, but containing quasi-spherical precipitates beyond the periphery of component B areas.
2. Subsequently, cells do not undergo phase reactions and only participate in diffusion exchange with the continuously transformed matrix, which, when undergoing progressive ordering, transforms into the structure consisting of alternating layers of (i) remaining partially disordered phase and (ii) 5:19 and 2:7 phases and, for samples sintered from fine powders, forms the lamellar Z phase. In this case, almost all reaction products are superstructures of the matrix hexagonal CaCu_5_-type lattice [20].
3. The transformation of continuous matrix massive, which continues during stepped aging, leads to the formation of final boundary hexagonal structure based on Cu-rich 1:5 phases and of 2:7 and 5:19 phases resideus.

Mechanisms of the formation of the ordered boundary hexagonal structure will be discussed in subsequent works.

### 4.2. Phases of Pseudo-Single-Crystal Samples in High-Coercivity State

The structural states of permanent magnet materials based on SmCo_5_ and NdFeB, which are logical from the viewpoint of metallurgy, allowed one, already at the earlier stages of investigations, to accurately list the main phases. In the case of the simple binary Sm–Co system, the majority of authors, after numerous attempts to relate results of unsuccessful heat treatments with eutectoid decomposition of the 1:5 phase, agree that the optimum composition of magnets must include a small amount of the Sm_2_Co_7_ phase along with the main phase. (In terms of the present consideration, we ignore nonmetallic impurities). 

When passing to NdFeB magnets and upon alloying the compositions for increasing their coercivity and Curie temperature, five-component and more-component systems are considered. As an example, we consider magnets prepared from the (Nd_0.85_Tb_0.15_)_16_(Fe_0.8_Co_0.2_)_76_B_8_ alloy with sufficiently simple composition. The list of phases for the aforementioned composition, according to experimental data, includes phases of concrete compositions, such as (Nd_0.8_Tb_0.2_)_2_(Fe_0.8_Co_0.2_)_14_B, (Nd_0.8_Tb_0.2_)_1_(Fe_0.8_Co_0.2_)_4_B_4_, (Nd_0.95_Tb_0.05_)_1_(Fe_0.5_Co_0.5_)_2_, and Nd_7_Co_3_.

In the majority of studies related to the (Sm,Zr)(Co,Cu,Fe)_z_ permanent magnets, lists of phases are almost absent. The chemical compositions of phases are given in the form of either tables of element contents or element balance dependences, in particular, for the heat-treatment stage. Usually, one has to make do with common designations of phases (1:5; 2:17, etc.), as they were shown above. 

Our opinion about the fact that such understatements are related to the formula representation of the integral alloy composition and its individual structural components and phases is available in our previous study [9]. 

In the present section, we attempt to present results obtained by EPMA of structural components of cast pseudo-single crystals in the high-coercivity state. It is possible to demonstrate the results most clearly using the standard coordinate grid of a three-component equilibrium diagram of metallic systems on the coordinates Sm–Zr–(Co,Cu,Fe).

**Figure 8:**
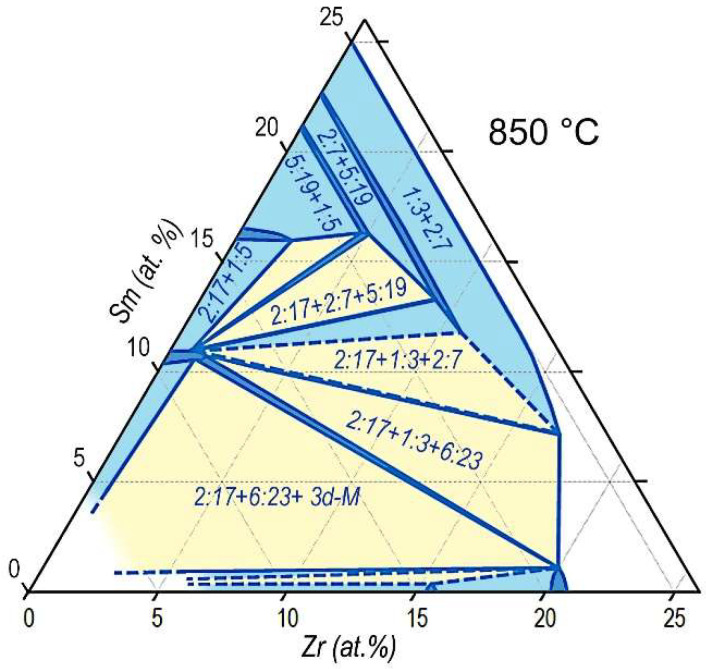
Isothermal section of the ternary Sm–Zr–Co system at 850 °С.

For the better understating of the given information, Figure 8 shows the isothermal section of the equilibrium ternary Sm–Zr–Co phase diagram at 850 °С, which is drawn using graphical and table data available in Derkaoui et al. [21]; the triangle sides are given in accordance with phase diagrams available in our previous study [9].

In turn, Table 3 and Table 4 give experimental data obtained in [1,2,3,4,5,6,7,8] for the high-coercivity phase compositions of (Sm,Zr)(Co,Cu,Fe)_z_; the data are available in [1,2,3,4,5,6,7,8] in the form of tables and dependencies.

Table 3 shows the compositions, for the convenient comparison, presented in the forms Sm(Co,Cu,Fe,Zr)_z_ and (Sm,Zr)(Co,Cu,Fe)_z_, if another representation is not given by authors (see, for example, the representation of the Zr-rich phase in [6]).

For Table 3 and Table 4, we selected the majority of available data for samples, the structural states of which were analyzed by authors [1,2,3,4,5,6,7,8] after heat treatment imitating the complete set of convenient heat treatments for the high-coercivity state of (Sm,Zr)(Co,Cu,Fe)_z_ permanent magnets.

Table 4 shows data characterizing the initial state of samples, aging conditions, and methods used for the analysis of phase compositions (which are given in authors editions [1,2,3,4,5,6,7,8]).

**Figure 9:**
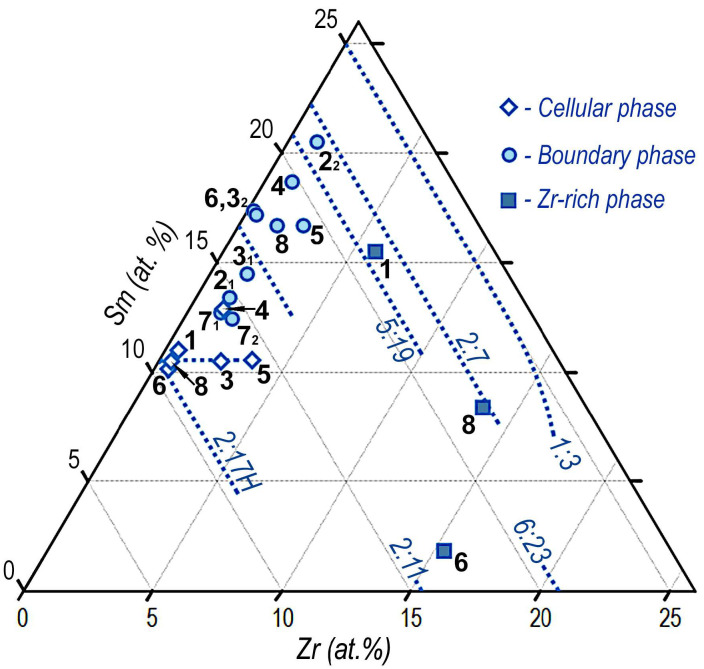
Visual presentation, on coordinates Sm–Zr–(Co,Cu,Fe), of the analysis data for the phase composition of high-coercivity (Sm,Zr)(Co,Cu,Fe)_z_ samples, which are available in [1,2,3,4,5,6,7,8] and given in Table 3 and Table 4. Numbers given near symbols correspond to Reference No. (see columns Ref. in Table 3 and Table 4).

The visualization of results of the analysis of the phase compositions (Table 3 and Table 4) is given on coordinates of the Sm–Zr–(Co,Cu,Fe) phase diagram in Figure 9. Each of the reported phases (cellular phase, boundary phase, and Zr-rich phase) is indicated by a corresponding symbol (in accordance with the legend in Figure 9). Digits given near symbols correspond to references in Table 3 and Table 4 (see columns Ref.). Dotted lines in Figure 9 indicate propagation of single-phase regions of the 2:17R and 2:17H, 1:5, 5:19, 2:7, 1:3, 2:11, and 6:23 phases.

The reported visualization of the experimental results of analysis of phase compositions in the form of both data taken from different studies and generalized data, at first sight, looks like “buck-shot” patterns.

Let us to briefly analyze the results.

Results of the analysis of the cellular phase available in Derkaoui et al. [1], Ray [8], and Xiong et al. [6] seemed to be more reliable and logical. As was found by many investigators, this phase is characterized by limited Cu and Zr contents, corresponds to a rhombus-like region with a slightly widened homogeneity range that originates from the quasi-ternary Sm_2_(TM)_17_ composition, and corresponds to formula Sm_2_(Co,Cu,Fe,Zr)_17_. According to data of Corte-Real et al. [3] and Goll et al. [5], symbols of the cellular phase are arranged within the same region, but the phase is characterized by substantially higher Zr contents. It is likely that, in this case, the Zr-rich phase that falls in the probe area affects the analysis results and distorts them. Data of Kronmuller and Goll [4] for the cellular phase seemed to be rather dubious; we obtained them by IT analysis of a dependence given in [4] rather than for concrete tabulated data.

Results of the analysis of the boundary phase are given in the form of a wide area of symbols from the center of the region between areas 2:17R and 1:5 to the center of region between 5:19 and 2:7. It should be noted that one of symbols does not fall into the (Sm,Zr)(TM)_5_ phase area. At the same time, data from Goll et al. [5] and Ray [8] adequately fall into the area of the 1:5 phase given in Figure 8.

It is difficult to form an opinion about the boundary phase based on the available data. However, the fact that the data of Corte-Real et al. [3] for the boundary phase at triple junctions correspond to data of Xiong et al. [6] should be noted. In this case, positions of symbols indicate the enrichment of the composition in samarium as compared to the 1:5 phase stoichiometry. This disagrees with the spinodal concept available in [6] and data of [17,22] on phases and phase reactions for the (Sm,Zr)(Co,Cu,Fe)_z_ alloys during aging.

Symbols corresponding to the Zr-rich phase were found to be scattered in the (Sm,Zr)_5_(TM)_19_–Sm,Zr)_2_(TM)_7_ two-phase [1] and (Sm,Zr)_2_(TM)_7_–(Sm,Zr)_2_(TM)_11_–(Sm,Zr)_6_(TM)_23_ three-phase [6] regions, and directly in the phase homogeneity region (Sm,Zr)_2_(TM)_7_ [8]; these data are difficult to explain.

**Figure 10:**
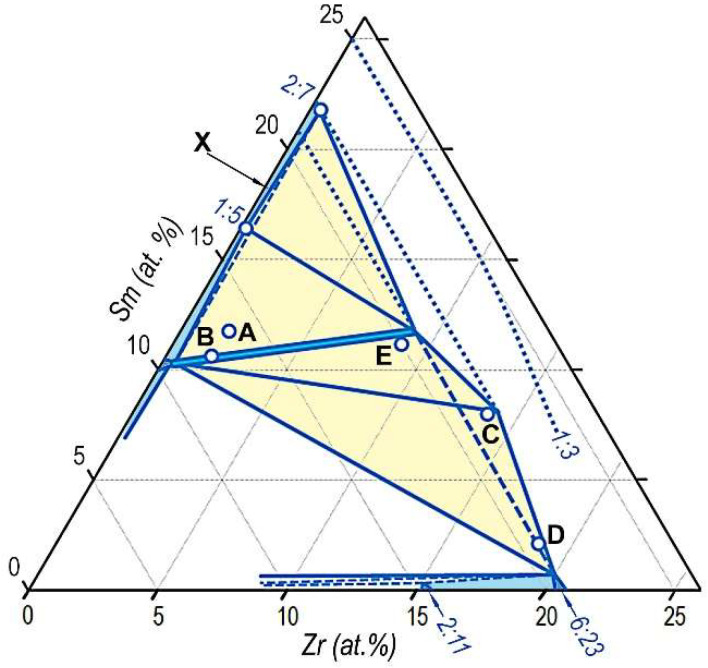
Visualization, on coordinates Sm-Zr-(Co, Cu, Fe), of results (Table 2) of analysis of phase compositions of the high-coercivity pseudo-single-crystal (Sm, Zr)(Co, Cu , Fe)z alloy samples.

Let us again consider the averaged data obtained in the present study for the pseudo-single-crystal samples in the high-coercivity states. The results of the analysis of the phase compositions of the high-coercivity (Sm,Zr)(Co,Cu,Fe)_z_ alloy series, which are available in Table 2, are visualized in Figure 10.

It should be noted that the formed images (Figure 10) obviously cannot be considered as the equilibrium phase diagram. The image is the visualization and is used by us to clearly represent the results obtained.

Taking into account the fact that structural components A and B are heterogeneous, their positions in Figure 10 are given for reference. In this case, attention should be paid to the fact that symbols corresponding to structural components A and B adequately fall on the right of the three-phase 2:17R–2:17H–1:5 triangle given in the sketch of the isothermal section (1160–1180 °С) of the 3d-TM angle of the sketch quasi-ternary (Co,Cu,Fe)– Sm–Zr phase diagram available in [9].

As is seen, the positions of phase components are sufficiently reasonable. Symbols of structural elements (Table 2) are arranged near corresponding dotted areas of assumed single-phase regions of the corresponding phases indicated in Figure 8 and Figure 9.

We have allowed ourselves, in Figure 10, to add probable interrelations of phases for a possible sketchy prototype of an equilibrium phase diagram, but with the persistent additional note that the structure of the samples studied is definitely metastable.

## 5. Conclusions

We have presented the original view on the issues of the structure formation of the (R,Zr)(Co,Cu,Fe)_z_ alloys in the concentration ranges promising for manufacturing high-coercivity permanent magnets. These findings are based on the analysis of the microstructures of the pseudo-single-crystal samples of these alloys in a high-coercivity state.

The following have been shown.

The structures are characterized by different levels of heterogeneity, which determines their magnetic hysteresis and, in particular, the coercivity mechanism consisting in the domain-wall pinning at structural “partitions”. At the optical magnification level (the first level of heterogeneity), the high-coercivity structure of (Sm,Zr)(Co,Cu,Fe)_z_ pseudo-single crystals is formed by three structural components A, B, and C; the relationship of the volume fractions of these structural components is determined by the integral chemical composition of alloys.

Within the studied ranges of chemical compositions of experimental Sm_1−x_Zr_x_(Co_1−a−b_Cu_a_Fe_b_)_z_ alloy series, the monotonic increase in each of the independent variables (X and Z) and mutual changes in dependent variables (a and b) lead to monotonic changes in the relationship of volume fractions of structural components A and B, namely, from V_A_ ≥ V_B_ to V_A_ ≤ V_B_.

At the level of scanning electron microscopy magnifications (second level of heterogeneity), the high-coercivity structure of the (Sm,Zr)(Co,Cu,Fe)_z_ alloys is represented by precipitates in the spatially continuous matrix, which is common for main structural components A and B. The structure of component B is formed by hexagonal bipyramids of the cellular phase interspersed into a continuous boundary matrix network. In turn, the structural component A is formed by thinner, modulated quasi-spherical precipitates interspersed into a continuous boundary matrix network; apparently, the structural component X (Sm(Co,Cu,Fe)_3.5-5_) is the result of the coalescence of these precipitates.

The maximum local coercivity of the domain wall is observed on coring A + B, where precipitates typical of both main structural components are interspersed in the matrix. Along with structural components A and B, Zr-rich phases, such as (Sm_0.56_Zr_0.44_)_5_(Co,Cu,Fe)_19_, (Sm_0.37_Zr_0.63_)_2_(Co,Cu,Fe)_7_, and (Zr_0.90_Sm_0.10_)_6_(Co,Cu,Fe)_23_ are found in the alloy structure within structural component A.

The magnetic performance of the samples is characterized by:

(i) almost-ultimate magnetic hysteresis loops (that correspond to conditions 4πJ_S_ = 4πJ_R_ and (BH)_MAX_ = (4πJ_S_)^2^/4) in the case of the dominant volume fraction of component A in the structure of samples (V_A_ ≥ V_B_);
(ii) intense increase in the coercive force (H_CJ_) and hysteresis loop squareness parameter (H_k_) as the volume fraction of structural component A decreases;
(iii) the progressive deceleration of the increase in H_CJ_ and reaching the saturation (“plateau”) and the extreme of H_k_ (with a maximum) as the volume fractions of structural components A and B in the alloys become equal (V_A_ = V_B_);
(iv) worsening the hysteresis loop squareness as the volume fraction of structural component A becomes lower than that of structural component B (V_A_ ≤ V_B_).

## Figures and Tables

**Table 1:** Chemical composition of Sm_1−x_Zr_x_(Co_1−a−b_Cu_a_Fe_b_)_z_ experimental series alloys.

Series No.	x	a	b	z
1	0.13	0.088	0.210	6.0–6.8
2	0.15	0.088	0.210	6.0–6.8
3	0.17	0.088	0.210	6.0–6.8
4	0.19	0.088	0.210	6.0–6.8
5	0.15	0.075	0.260	6.0–6.8
6	0.13	0.070	0.240	6.1–6.5

**Table 2:** Averaged chemical compositions of structural components A and B and phases C, D, and E and structural component X, which are present only within component A.

Structural Component	Composition (Version 1)	Composition (Version 2)	Main Phases
A	Sm(Co_0.69_Cu_0.10_Fe_0.19_Zr_0.02_)_7.5_	Sm_0.86_Zr_0.14_(Co_0.70_Cu_0.10_Fe_0.20_)_6.3_	1:5
B	Sm(Co_0.69_Cu_0.07_Fe_0.22_Zr_0.02_)_8.4_	Sm_0.86_Zr_0.14_(Co_0.70_Cu_0.07_Fe_0.23_)_7.1_	2:17
C	Sm(Co_0.67_Cu_0.04_Fe_0.14_Zr_0.15_)_11.6_	Sm_0.37_Zr_0.63_(Co_0.78_Cu_0.05_Fe_0.17_)_3.6_	2:7
D	Sm(Co_0.62_Cu_0.02_Fe_0.17_Zr_0.19_)_46.2_	Sm_0.10_Zr_0.90_(Co_0.76_Cu_0.03_Fe_0.21_)_3.8_	6:23
E	Sm(Co_0.68_Cu_0.07_Fe_0.15_Zr_0.10_)_8.0_	Sm_0.56_Zr_0.44_(Co_0.76_Cu_0.08_Fe_0.16_)_4.0_	5:19
X	Sm(Co_0.50–0.63_Cu_0.22–0.40_Fe_0.10–0.15_)_3.5–5.0_		2:7–1:5

**Table 3:** Experimental data for the integral compositions and individual phases of the high-coercivity (Sm,Zr)(Co,Cu,Fe)z alloys [1,2,3,4,5,6,7,8].

Ref.	Alloy composition	Cellular Phase	Boundary Phase	Zr-Rich Phase
[1]	Sm(Co_0.69_Cu_0.06_ Fe_0.22_Zr_0.03_)_7.2_ Sm_0.82_Zr_0.18_(Co_0.71_Cu_0.06_Fe_0.23_)_5.74_	Sm(Co_0.68_Cu_0.04_Fe_0.28_Zr_0.006_)_8.09_ Sm_0.96_Zr_0.04_(Co_0.68_Cu_0.04_Fe_0.28_)_7.70_	-	Sm(Co_0.68_Cu_0.11_Fe_0.14_Zr_0.07_)_5.49_ Sm_0.72_Zr_0.28_(Co_0.73_Cu_0.12_Fe_0.15_)_3.67_
[2]_1_	Sm(Co_0.898_Cu_0.06_Fe_0.015_Zr_0.027_)_6.4_ Sm_0.85_Zr_0.15_(Co_0.923_Cu_0.062_Fe_0.015_)_5.3_	-	Sm(Co_0.61_Cu_0.17_Fe_0.205_Zr_0.015_)_6.46_ Sm_0.91_Zr_0.09_(Co_0.62_Cu_0.17_Fe_0.21_)_5.80_	-
[2]_2_			Sm(Co_0.474_Cu_0.37_Fe_0.142_Zr_0.014_)_3.88_* Sm_0.95_Zr_0.05_(Co_0.48_Cu_0.38_Fe_0.14_)_3.63_*	
[3]_1_	Sm(Co_0.80_Cu_0.108_Fe_0.06_Zr_0.03_)_7.20_ Sm_0.81_Zr_0.19_(Co_0.83_Cu_0.11_Fe_0.06_)_5.63_	Sm(Co_0.87_Cu_0.03_Fe_0.07_Zr_0.03_)_8.52_ Sm_0.81_Zr_0.19_(Co_0.89_Cu_0.03_Fe_0.08_)_6.75_	Sm(Co_0.69_Cu_0.244_Fe_0.05_Zr_0.016_)_5.90_ Sm_0.91_Zr_0.09_(Co_0.70_Cu_0.25_Fe_0.05_)_5.29_	-
[3]_2_			Sm(Co_0.51_Cu_0.466_Fe_0.02_Zr_0.004_)_4.78_* Sm_0.98_Zr_0.02_(Co_0.51_Cu_0.47_Fe_0.02_)_4.68_*	
[4]	Sm(Co_0.67_Cu_0.07_Fe_0.22_Zr_0.04_)_7.4_ Sm_0.77_Zr_0.23_(Co_0.70_Cu_0.07_Fe_0.23_)_5.48_	Sm(Co_0.71_Cu_0.015_Fe_0.26_Zr_0.015_)_6.7_ Sm_0.91_Zr_0.09_(Co_0.72_Cu_0.02_Fe_0.26_)_6.0_	Sm(Co_0.58_Cu_0.30_Fe_0.107_Zr_0.013_)_4.35_ Sm_0.95_Zr_0.05_(Co_0.58_Cu_0.31_Fe_0.11_)_4.06_	-
[5]	Sm(Co_0.67_Cu_0.07_Fe_0.22_Zr_0.04_)_7.4_ Sm_0.77_Zr_0.23_(Co_0.70_Cu_0.07_Fe_0.23_)_5.48_	Sm(Co_0.69_Cu_0.02_Fe_0.25_Zr_0.04_)_8.5_ Sm_0.75_Zr_0.25_(Co_0.72_Cu_0.02_Fe_0.26_)_6.09_	Sm(Co_0.56_Cu_0.29_Fe_0.12_Zr_0.03_)_5.0_ Sm_0.87_Zr_0.13_(Co_0.58_Cu_0.30_Fe_0.12_)_4.22_	-
[6]	Sm(Co_0.72_Cu_0.055_Fe_0.20_Zr_0.025_)_7.5_ (Sm_0.84_Zr_0.16_)(Co_0.74_Cu_0.06_Fe_0.21_)_6.2_	Sm(Co_0.76_Cu_0.016_Fe_0.22_Zr_0.007_)_8.8_ Sm_0.94_Zr_0.06_(Co_0.76_Cu_0.02_Fe_0.22_)_8.2_	Sm(Co_0.64_Cu_0.23_Fe_0.13_Zr_0.004_)_4.7_ Sm_0.98_Zr_0.02_(Co_0.64_Cu_0.23_Fe_0.13_)_4.6_	Sm_0.08_Zr_0.67_Cu_0.25_(Co_0.89_Fe_0.11_)_3.3_** Sm_0.11_Zr_0.89_(Co_0.83_Cu_0.07_Fe_0.10_)_4.8_
[7]_1_	Sm(Co_0.805_Cu_0.08_Fe_0.10_Zr_0.015_)_8.5_ Sm_0.89_Zr_0.11_(Co_0.82_Cu_0.08_Fe_0.10_)_7.43_	-	Sm(Co_0.70_Cu_0.205_Fe_0.08_Zr_0.015_)_6.87_ Sm_0.91_Zr_0.09_(Co_0.71_Cu_0.21_Fe_0.08_)_6.14_	-
[7]_2_	Sm(Co_0.78_Cu_0.08_Fe_0.10_Zr_0.04_)_8.5_ Sm_0.75_Zr_0.25_(Co_0.81_Cu_0.08_Fe_0.11_)_6.09_	-	Sm(Co_0.66_Cu_0.25_Fe_0.07_Zr_0.02_)_7.06_ Sm_0.87_Zr_0.13_(Co_0.67_Cu_0.26_Fe_0.07_)_5.99_	-
[8]	Sm(Co_0.67_Cu_0.08_Fe_0.23_Zr_0.02_)_8.52_ Sm_0.85_Zr_0.15_(Co_0.68_Cu_0.082_Fe_0.24_)_7.13_	Sm(Co_0.69_Cu_0.05_Fe_0.25_Zr_0.006_)_8.51_ Sm_0.95_Zr_0.05_(Co_0.70_Cu_0.05_Fe_0.25_)_8.02_	Sm(Co_0.41_Cu_0.52_Fe_0.06_Zr_0.02_)_5.0_ Sm_0.92_Zr_0.08_(Co_0.41_Cu_0.53_Fe_0.06_)_4.5_	Sm(Co_0.65_Cu_0.06_Fe_0.15_Zr_0.15_)_10.9_ Sm_0.38_Zr_0.62_(Co_0.76_Cu_0.07_Fe_0.19_)_3.54_

*-boundary in the triple junction of cells. **-in the author’s edition. [2]_1_, [2]_2_, [3]_1_, [3]_2_, [7]_1_, [7]_2_–according to authors’ data, chemical composition variations.

**Table 4:** Initial states, heat treatment conditions, and experimental methods used for the study of alloys listed in Table 3.

Ref.	Sample Initial State	Aging Heat Treatment Parameters			Study Method
		Isothermal, T, °C/time, h	Cooling Rate, °C/min	Final T, °C	
[1]	As-cast	950°/45 h + 850°/300 h	0.0625	400°	EPMA
[2]	Sintered	700°/24 h	0.5–1	400°	TEM/Nanoprobe
[3]	Sintered	790°/36 h	0.7	400°	TEM/Nanoprobe
[4]	Sintered	800°/16 h	1	400°	TEM-EDX
[5]	Sintered	800°/16 h	1	400°	TEM-EDX
[6]	Sintered	820°/6 h	0.5	520°	3DAP
[7]	As-cast	850°/12 h	0.7	400°	TEM/Nanoprobe
[8]	Sintered and As-cast	800°/2–72 h	?	?	AEM/SEM-EDX

## Data Availability

The data presented in this study are openly available.
